# Brainstem abscess of undetermined origin: microsurgical drainage and brief antibiotic therapy

**DOI:** 10.1590/1516-3180.2014.1322635

**Published:** 2014-04-01

**Authors:** Pedro Tadao Hamamoto, Marco Antonio Zanini

**Affiliations:** I MD. Medical Resident, Department of Neurology, Psychology and Psychiatry, Faculdade de Medicina de Botucatu (FMB), Universidade Estadual Paulista (Unesp), Botucatu, São Paulo, Brazil; II MD, PhD. Associate Professor, Department of Neurology, Psychology and Psychiatry, Faculdade de Medicina de Botucatu (FMB), Universidade Estadual Paulista (Unesp), Botucatu, São Paulo, Brazil

**Keywords:** Brain stem, Abscess, Central nervous system bacterial infections, Neurosurgical procedures, Anti-bacterial agents, Tronco encefálico, Abscesso, Infecções bacterianas do sistema nervoso central, Procedimentos neurocirúrgicos, Antibacterianos

## Abstract

**CONTEXT:**

Solitary brainstem abscesses are rare and they are usually associated with other infections. They are severe conditions with high morbidity and mortality. The surgical options are stereotactic aspiration and microsurgical drainage. Systemic antibiotic therapy is used for more than six weeks.

**CASE REPORT:**

We present the case of a young man with a solitary abscess at the pons, without other systemic infections. The patient was treated by means of microsurgical drainage and antibiotic therapy for three weeks. His postoperative recovery was good.

**CONCLUSIONS:**

A microsurgical approach may be considered to be an important option for large abscesses that are multiloculated, close to the surface or contain thick fluid. Complete emptying of the purulent accumulation may diminish the required duration of antibiotic therapy.

## Abstract (pt)

**CONTEXTO:**

Abscessos isolados do tronco encefálico são raros e geralmente associados a outras infecções. Trata-se de condição grave, com grande morbidade e mortalidade. Opções cirúrgicas são aspiração com estereotaxia e drenagem microcirúrgica. Antibioticoterapia sistêmica tem sido usada por mais de seis semanas.

**RELATO DE CASO:**

Apresentamos o caso de um jovem com abscesso pontino sem outras infecções sistêmicas. O paciente foi tratado com drenagem microcirúrgica e antibioticoterapia por três semanas. Houve boa evolução pós-operatória.

**CONCLUSÕES:**

Acesso microcirúrgico pode ser considerado uma opção importante no tratamento de grandes abscessos do tronco encefálico, que são multiloculados, próximos da superfície ou que contenham líquido espesso. Drenagem completa do material purulento pode diminuir o período de antibioticoterapia.

## INTRODUCTION

Solitary brainstem abscesses of undetermined origin are quite rare and account for less than 1% of all intracranial abscesses.^1^ ^,^ ^2^ Before the advent of accurate imaging techniques, they were typically diagnosed at autopsy.^3^ ^,^ ^4^ Mortality was very high, and surgery would be prohibitive. The development of computed tomography and magnetic resonance imaging, improvements in surgical techniques and broad-spectrum antibiotics have improved the prognosis.^5^

Except for their unique vital location, many features of brainstem abscesses are common to all cerebral abscesses. Surgical management and broad-spectrum antibiotic therapy favor good functional recovery.^6^ We present a case of a solitary abscess at the pons, of unknown etiology. 

## CASE REPORT

**Figure 1:**
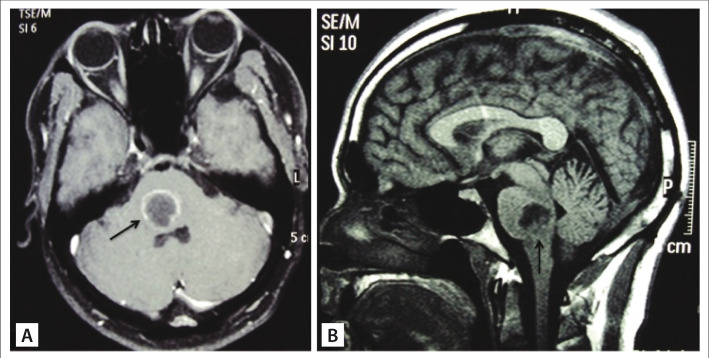
Magnetic resonance imaging showing low T1 signal, with ring enhancement at the dorsal pars of the pons (arrows). (A - axial view; B - sagittal view)

A 27-year-old male farmworker was referred to our service with a 10-day history of progressive headache, gait disturbance, diplopia, right facial numbness and weakness of the left arm and leg. He had no fever and no evidence of systemic infections such as endocarditis, sinusitis or otitis. A neurological examination showed ataxic gait, right dysmetria, left hemiparesis, dysarthria, right abducens nerve palsy and right facial nerve palsy. His laboratory tests and serological tests were normal. A cerebrospinal fluid examination was unremarkable. All further investigations for other infection sites were negative. Magnetic resonance imaging showed low T1 signal with ring enhancement after gadolinium administration, located at the right dorsal tegmentum of the pons, thus suggesting the presence of an abscess or a focal glioma (Figure 1). Restricted diffusion, seen on diffusion-weighted imaging, favored the hypothesis of an abscess. Suboccipital craniotomy was performed, and the floor of fourth ventricle was exposed using a telovelar approach. There was gentle bulging of the entire rhomboid fossa, and no floating point could be detected. First, puncturing was performed, and 3 cm^3^ of purulent yellowish fluid was drained. Gentle opening of the drainage point (1 mm) enabled more copious and spontaneous drainage. No hard capsule was detected. When the output of pus ceased and the tension on the rhomboid floor decreased, the surgical procedure was terminated. Broad-spectrum antibiotics were immediately administered (oxacillin, ceftriaxone and metronidazole) and were maintained for three weeks. Culturing was negative.

**Figure 2.**
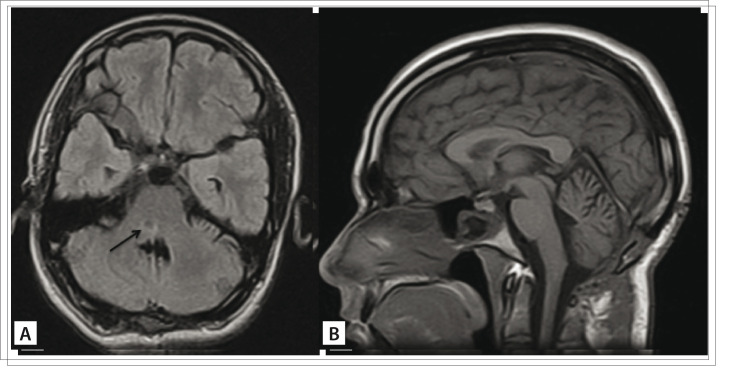
Magnetic resonance imaging produced in the sixth postoperative month, showing mild hypersignal within the pons (arrow), through FLAIR (fluid-attenuated inversion recovery) acquisition (A, axial view). No enhancement is seen with T1 acquisition (B, sagittal view)

The early progress after surgery was uneventful, without any additional deficits. At the six and ten-month follow-ups, the patient presented improvement of all previous symptoms but continued to have mild hemiparesis and facial palsy. Magnetic resonance imaging produced in the sixth postoperative month showed only a mild hypersignal within the pons, from fluid-attenuated inversion recovery acquisition (Figure 2).

## DISCUSSION

Brainstem abscesses are uncommon and cases with solitary brainstem abscesses of undetermined etiology are even rarer.^1^ ^,^ ^4^ They are often associated with immune disorders such as HIV infection and diabetes.^7^ The clinical presentation depends on the abscess size and the stage of infection.6 Even small abscesses can cause significant clinical deficits because the space is limited.6 Large abscesses can cause hydrocephalus secondary to fourth-ventricle obstruction.^4^

Depending on the aggressiveness of the microorganism involved and on the patient's immunological condition, an infection may be extremely destructive and cause rapid clinical deterioration. The most common causative microorganisms identified are *Streptococcus spp*. and *Staphylococcus spp*.^8^

Computed tomography scans show an irregular low-density lesion and, after contrast injection, observation of an enhanced-ring sign suggests the presence of a focal and/or cystic lesion, which is difficult to differentiate from other lesions. Magnetic resonance imaging is more sensitive and specific for the diagnosis. In the early phases of brain abscess development, an irregular, enhanced low signal suggests cerebritis. In the late stages, the vascularized capsule shows a ring-contrasted lesion. Using diffusion-weighted imaging, a restricted pattern can be observed. Spectroscopy can be useful for identifying a causative microorganism.^9^

The best treatment for a solitary brainstem abscess of undetermined origin has yet to be determined,^10^ but it currently includes conservative management with systemic antibiotics, microsurgery or stereotactic aspiration. In the early stages of development, or for small abscesses (< 2 cm), empirical treatment with broad-spectrum antibiotics must be started promptly and maintained for an extended period of time.^8^ ^,^ ^10^ Close monitoring of the patient's neurological condition and imaging findings is mandatory. If clinical deterioration or an increase in the abscess size occurs, changes to the antibiotics administered and surgical treatment should be considered. The surgical options for abscess treatment include stereotactic aspiration or microsurgical evacuation.^7^ While stereotactic aspiration is a minimally invasive technique, it may be difficult to drain thick or multiloculated abscesses.^1^ Although brainstem hemorrhage has low rates of occurence,^11^ it may be hazardous and uncontrolled during stereotactic surgery.

Direct microsurgical approaches result in greater drainage volume, even for multiloculated abscesses or content of greater viscosity. Using direct microsurgical approaches, a surgeon can also control for occasional bleeding and view abscess decompression. In large abscesses (> 2 cm) with organized capsules, poor clinical condition and uncertain diagnosis, surgery should be considered to be the first-line treatment. If the abscess is close to the surface of the brainstem, a surgical approach seems reasonable. In contrast, deep-seated small lesions would only require medication treatment, regardless of the clinical condition.

If the causative microorganism can be identified, a directed antibiotic scheme can be implemented. However, up to one third of brainstem abscesses show negative cultures.^11^ When the causative microorganism cannot be identified, empirical antibiotic therapy should be administered. The duration of antibiotic therapy is unclear. Most published reports continued the treatment for six to eight weeks, or more.^4^ ^,^ ^6^ ^,^ ^8^ ^-^ ^10^ ^,^ ^12^ In the present case, an abscess localized in the pons was drained using a suboccipital telovelar approach. Suction of the purulent content was insufficient to reduce the bulging. After a small incision was made at the location of the puncture, the drainage became more copious and much greater abscess shrinkage was seen. Antibiotic treatment was started after the surgical procedure and was maintained for three weeks. We believe that the full drainage of the abscess and optimal viewing of its collapse enabled a shorter period of antibiotic therapy.

We searched for similar cases in different databases (PubMed, Lilacs and Embase) using the terms: "brainstem" AND "abscess" AND "surgery", limited to "human" (Table 1). We found that few cases have been published. Abstracts or full texts were analyzed and it was seen that less than 30 reports were similar to ours. Moreover, only one of these published cases was from Brazil.^13^

**Table 1.** Results from our literature review of medical databases for case reports and case series of brainstem abscesses that were treated surgically. Search conducted on February 14, 201

Database	Search strategies	Papers found	Papers related
Medline (via PubMed)	(((brain stem) AND abscess) AND surgery) AND “case reports” [Publication Type]	88	14
Embase (via Elsevier)	(((brain stem) AND abscess) AND surgery) AND “case reports” [Publication Type]	47	11
Lilacs	abscesso [Palavras]ANDtronco [Palavras]ANDhumanos [Limites]	8	2

## CONCLUSION

A microsurgical approach may be considered to be an important option for large abscesses that are multiloculated, close to the surface or contain thick fluid. Complete emptying of the purulent accumulation may diminish the required duration of antibiotic therapy.

## References

[1] Arzoglou V, D'Angelo L, Koutzoglou M, Di Rocco C (2011). Abscess of the medulla oblongata in a toddler: case report and technical considerations based on magnetic resonance imaging tractography. Neurosurgery.

[2] Nathoo N, Nadvi SS, Narotam PK, van Dellen JR (2011). Brain abscess: management and outcome analysis of a computed tomography era experience with 973 patients. World Neurosurg.

[3] Danziger J, Allen KL, Bloch S (1974). Brain-stem abscess in childhood. Case report. J Neurosurg.

[4] Jamjoom ZA (1992). Solitary brainstem abscess successfully treated by microsurgical aspiration. Br J Neurosurgery.

[5] Rosenblum ML, Hoff JT, Norman D, Weinstein PR, Pitts L (1978). Decreased mortality from brain abscesses since the advent of computerized tomography. J Neurosurg.

[6] Wait SD, Beres EJ, Nakaji P (2009). Bacterial abscess of the medulla oblongata. J Clin Neurosci.

[7] Kirchhoff DC, Kirchhoff DFB, Muoio V (2008). Abscessos do tronco cerebral: apresentação de seis casos [Brain stem abscess: a study of six cases]. Arq Bras Neurocir.

[8] Fulgham JR, Wijdicks EF, Wright AJ (1996). Cure of a solitary brainstem abscess with antibiotic therapy: case report. Neurology.

[9] Lai PH, Li KT, Hsu SS (2005). Pyogenic brain abscess: findings from in vivo 1.5-T and 11.7-T in vitro proton MR spectroscopy. AJNR Am J Neuroradiol.

[10] Ghannane H, Laghmari M, Aniba K, Lmejjati M, Benali SA (2011). Diagnostic and management of pediatric brain stem abscess, a case-based update. Childs Nerv Syst.

[11] Suzer T, Coskun E, Cirak B, Yagci B, Tahta K (2005). Brain stem abscesses in childhood. Childs Nerv Syst.

[12] Nakajima H, Iwai Y, Yamanaka K, Kishi H (1999). Successful treatment of brainstem abscess with stereotactic aspiration. Surg Neurol.

[13] Hermes de N Jr, Rodrigues Pereira EL, Castro Ribeiro DE (2011). Staphylococcus aureus brainstem abscess in a Brazilian Amazon man. Case report. J Neurosurg Sci.

